# Association between depression and anxiety and adverse obstetric and neonatal outcomes: A retrospective National Inpatient Sample database study

**DOI:** 10.1097/MD.0000000000050353

**Published:** 2026-08-28

**Authors:** Fufei Zhang, Xinran Zhao, Xinlin Huang, Jingyi Huang, Qinfeng Yang, Xuegao Yu, Xiaomin Hou, Mengning Dong

**Affiliations:** a School of Health, Dongguan Polytechnic, Dongguan, Guangdong, China; b The Second School of Clinical Medicine, Southern Medical University, Guangzhou, Guangdong, China; c School of Pharmaceutical Sciences, Southern Medical University, Guangzhou, Guangdong, China; d Division of Orthopaedic Surgery, Department of Orthopaedics, Nanfang Hospital, Southern Medical University, Guangzhou, Guangdong, China; e Department of Laboratory Medicine, The First Affiliated Hospital, Sun Yat-sen University, Guangzhou, Guangdong, China; f Department of Anesthesiology, Nanfang Hospital, Southern Medical University, Guangzhou, Guangdong, China.

**Keywords:** anxiety, depression, National Inpatient Sample, neonatal outcomes, obstetric outcomes

## Abstract

Maternal depression and anxiety are common perinatal conditions; however, large-scale, nationally representative evidence on their association with a comprehensive range of obstetric and neonatal outcomes remains limited. The study utilized the International Classification of Diseases, 9th and 10th Revisions, Clinical Modification (ICD-9-CM and ICD-10-CM) to identify diagnostic codes for depression, anxiety, and obstetric outcomes. Data from the National Inpatient Sample were analyzed. Categorical variable differences were assessed via the chi-square test. Univariate and multivariate regression analyses were conducted to evaluate the relationship between maternal depression and anxiety and obstetric outcomes, with forest plots displaying odds ratios and 95% confidence intervals. A total of 6815,408 birth hospitalizations were included, with 323,705 women diagnosed with depression or anxiety. Multivariable analysis showed that birth hospitalizations with a documented diagnosis of depression or anxiety had higher odds of several adverse obstetric and neonatal outcomes, including malformation, severe preeclampsia, chorioamnionitis, major puerperal infection, intrauterine death, placenta previa, premature rupture of membranes, postpartum hemorrhage, fetal growth restriction, endometritis, uterine rupture, placental abruption, and preterm birth. These findings suggest that depression and anxiety diagnoses during birth hospitalization are associated with several adverse obstetric and neonatal outcomes. Maternal mental health history should be considered when assessing perinatal risk and planning care. The results also support continued screening, brief intervention, and referral for mental health concerns during prenatal and postpartum care. Future prospective studies with predelivery mental health assessment are needed to better evaluate the temporal sequence and potential causal pathways.

## 1. Introduction

Mental illnesses constitute a significant portion of the global burden of health-related issues.^[1]^ According to the Global Burden of Diseases, Injuries, and Risk Factors Study (GBD) conducted in 2019, depression and anxiety disorders were identified as the most debilitating mental health conditions. These 2 disorders were also among the top 25 contributors to the global health burden that year.^[2,3]^ According to information from the World Health Organization, mental health conditions like depression and anxiety are widespread, impacting around 4.4% and 3.6% of people worldwide, respectively.^[4–6]^ As of 2015, depression impacted over 322 million individuals globally, while anxiety affected 264 million. In the United States alone, the rates of depression and anxiety stood at 5.9% and 6.3%, corresponding to patient populations surpassing 17 million and 18 million, respectively.^[7]^ Some studies have shown that with the development of the times and the gradual increase in economic and other pressures, the problems of depression and anxiety have become increasingly serious.^[8]^

The increasing prevalence of depression and anxiety has been linked to negative outcomes following some surgical procedures.^[9]^ In obstetric care, depression is a prevalent complication during both pregnancy and the childbearing years. Major depressive disorder, as defined by diagnostic criteria during pregnancy, affects 12.7% of expectant mothers, and up to 37% of women report experiencing depressive symptoms at some point throughout their pregnancy.^[10]^ Anxiety is more commonly observed than depression at all stages of pregnancy, although the 2 disorders often co-occur, with a comorbidity rate of approximately 60%.^[10,11]^ In many of these studies, depression and anxiety are often intertwined, making it challenging to identify pregnant women who experience high levels of anxiety without also showing signs of depression.^[12]^

At present, the evidence regarding the precise impact of perinatal depression and anxiety on birth outcomes is still undefined and sometimes contradictory.^[13]^ Evidence from nationally representative sources remains limited on how depression and anxiety diagnoses during birth hospitalizations are associated with a broad range of obstetric and neonatal outcomes when analyzed within a unified framework. Using 10 years of National Inpatient Sample (NIS) data (2010–2019), this study assessed temporal trends in these diagnoses among birth hospitalizations and examined their adjusted associations with multiple outcome domains, including hypertensive, infectious, hemorrhagic, placental, fetal growth, and neonatal outcomes.

## 2. Materials and methods

### 2.1. Data source

The NIS database, which is supported by the Agency for Healthcare Research and Quality, represents the largest inpatient dataset in the United States. It is structured to provide nationally representative, weighted estimates of hospital discharges, capturing over 7 million hospital admissions annually. The data includes both primary and secondary diagnoses, medical procedures, discharge status, as well as demographic and clinical variables. Beginning in 2012, the NIS was redesigned to improve national estimates; within each stratum, a 20% stratified random sample of discharges from all Healthcare Cost and Utilization Project participating hospitals was drawn by the Agency for Healthcare Research and Quality.^[14]^ The data account for 20% of hospital admissions in the United States and cover more than 96% of the country’s population geographically.^[15]^ Because the NIS contains de-identified, publicly available administrative data, this study was exempt from institutional review board review. Informed consent was not required. This study complied with the Healthcare Cost and Utilization Project Data Use Agreement.

### 2.2. Study population

The analysis included pregnant women who were hospitalized for birth between 2010 and 2019. Diagnostic and procedure codes (Table S1, Supplemental Digital Content 1) from the International Classification of Diseases, 9th Revision, Clinical Modification (ICD-9-CM) and ICD-10-CM were used to identify a total of 7002,841 birth hospitalizations from 2010 to 2019.^[16]^ Pregnant women under 12 years of age, those with abortive pregnancies, and those with missing demographic characteristics or hospital information were excluded.

### 2.3. Exposure

**Figure 1.**
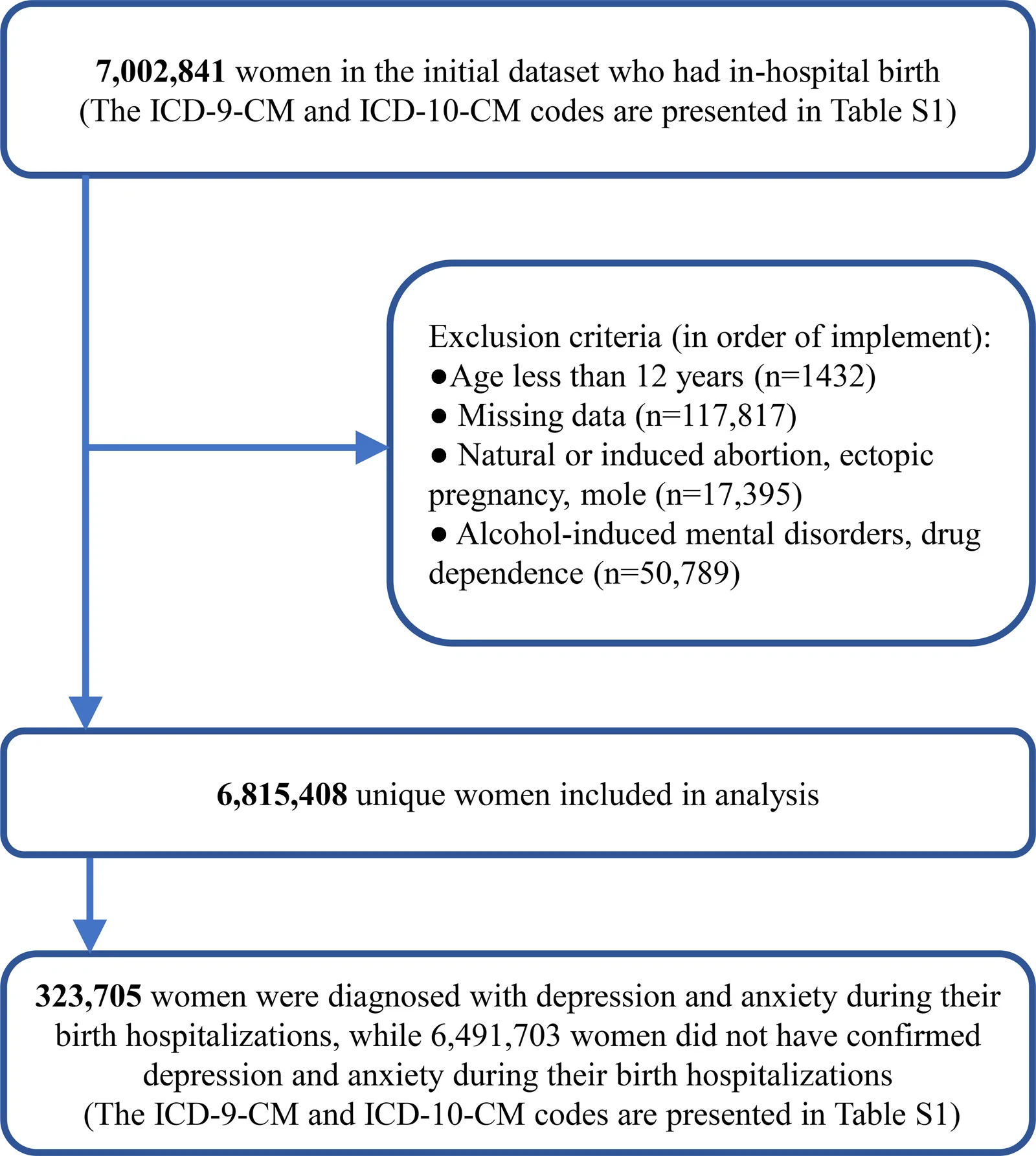
Flow and inclusion/exclusion of all women.

We used the ICD-9-CM and ICD-10-CM codes from the same birth hospitalization to identify depression and anxiety diagnoses (Table S1, Supplemental Digital Content 1). Although depression and anxiety were identified using separate code lists, the main analysis combined them into a single depression and anxiety group, defined as any birth hospitalization with either diagnosis. Because the NIS does not provide the timing or onset of these diagnoses, they should be interpreted as documented mental health diagnoses during birth hospitalization. Women with diagnosis codes for depression or anxiety were included. Additionally, 50,789 women with drug dependence or alcohol-induced psychiatric disorders were excluded to minimize confounding by substance use. In total, 6815,408 women with birth hospitalizations were included (Fig. 1). A validation study reported a sensitivity of 91% and a positive predictive value of 91% for this set of diagnostic codes.^[17]^ These women were categorized into 2 groups: those with depression or anxiety (n = 323,705) and those without (n = 6491,703).

### 2.4. Obstetric and neonatal outcomes

The obstetric and neonatal outcomes identified using ICD-9-CM or ICD-10-CM codes included antepartum hemorrhage, postpartum hemorrhage, preterm birth, intrauterine death, placenta previa, placenta accreta spectrum, placental abruption (PA), premature rupture of membranes (PROM), fetal distress, severe preeclampsia, excessive fetal growth, fetal growth restriction (FGR), major puerperal infection, malformation (fetal central nervous system malformation, chromosomal abnormalities, and maternal genetic disorders of the fetus), uterine rupture, chorioamnionitis, endometritis, and death (Table S2, Supplemental Digital Content 2). Maternal death during hospitalization was directly extracted from the NIS.

### 2.5. Covariates of interest

Covariates included demographic characteristics (age, race/ethnicity, length of stay), hospital information (type of admission, bed size of hospital, teaching status of hospital, location of hospital, type of insurance, total charge), The number of comorbidities including acquired immune deficiency syndrome, alcohol abuse, deficiency anemias, rheumatoid arthritis/collagen vascular diseases, chronic blood loss anemia, congestive heart failure, chronic pulmonary disease, coagulopathy, uncomplicated diabetes, diabetes with chronic complications, hypertension (combined uncomplicated and complicated), hypothyroidism, liver disease, lymphoma, fluid and electrolyte disorders, metastatic cancer, other neurological disorders, obesity, paralysis, peptic ulcer disease excluding bleeding, peripheral vascular disorders, psychoses, pulmonary circulation disorders, renal failure, solid tumor without metastasis, valvular disease, and weight loss. Demographic information and hospital characteristics were obtained directly from the NIS database. Additional variables were determined using discharge diagnoses and procedural coding.

### 2.6. Statistical analyses

Statistical analyses were conducted using SPSS version 25. To evaluate potential differences between the baseline clinical and demographic characteristics of the 2 study groups, the Wilcoxon rank-sum test (continuous variables) and the Chi-square test (categorical variables) were employed. Multivariate logistic regression models were constructed, adjusting for potential confounding factors including maternal demographics, preexisting clinical conditions, and co-occurring factors. The relationship between depression and anxiety and eighteen adverse obstetric and neonatal outcomes was explored by calculating odds ratios (OR) and 95% confidence intervals (CIs). All variables from the NIS, such as demographics, hospital characteristics, and preoperative comorbidities, were incorporated into the regression analysis (Table 1). Statistical significance was defined by an alpha level of *P* ≤ .001 due to the large sample size used in previous studies of the NIS.^[18]^

**Table 1:** Variables used in binary logistic regression analysis.

Variables categories	Specific variables
Patient demographics	Age (≥12 yr), race (White, Black, Hispanic, Asian or Pacific Islander, Native American and Other).
Hospital characteristics	Type of admission (nonelective, elective), bed size of hospital (small, medium, large), teaching status of hospital (nonteaching, teaching), location of hospital (rural, urban), type of insurance (Medicare, Medicaid, private insurance, self-pay, no charge, other), location of the hospital (northeast, Midwest or north central, south, west).
Comorbidities	AIDS, alcohol abuse, deficiency anemia, rheumatoid diseases, chronic blood loss anemia, congestive heart failure, chronic pulmonary disease, coagulopathy, diabetes (uncomplicated), diabetes (with chronic complications), drug abuse, hypertension, hypothyroidism, liver disease, lymphoma, fluid and electrolyte disorders, metastatic cancer, neurological disorders, obesity, paralysis, peripheral vascular disorders, psychoses, pulmonary circulation disorders, renal failure, solid tumor without metastasis, peptic ulcer disease, valvular disease, weight loss, multiple pregnancy.
Postpartum outcomes	Antepartum hemorrhage, postpartum hemorrhage, preterm birth, intrauterine death, placenta previa, placenta accreta spectrum, placental abruption, premature rupture of membranes, fetal distress, severe preeclampsia, excessive fetal growth, fetal growth restriction, major puerperal infection, malformation, uterine rupture, chorioamnionitis, endometritis, death.

AIDS = acquired immunodeficiency syndrome, LOS = length of stay.

## 3. Results

### 3.1. Occurrence of depression and anxiety in women undergoing birth hospitalizations

**Figure 2.**
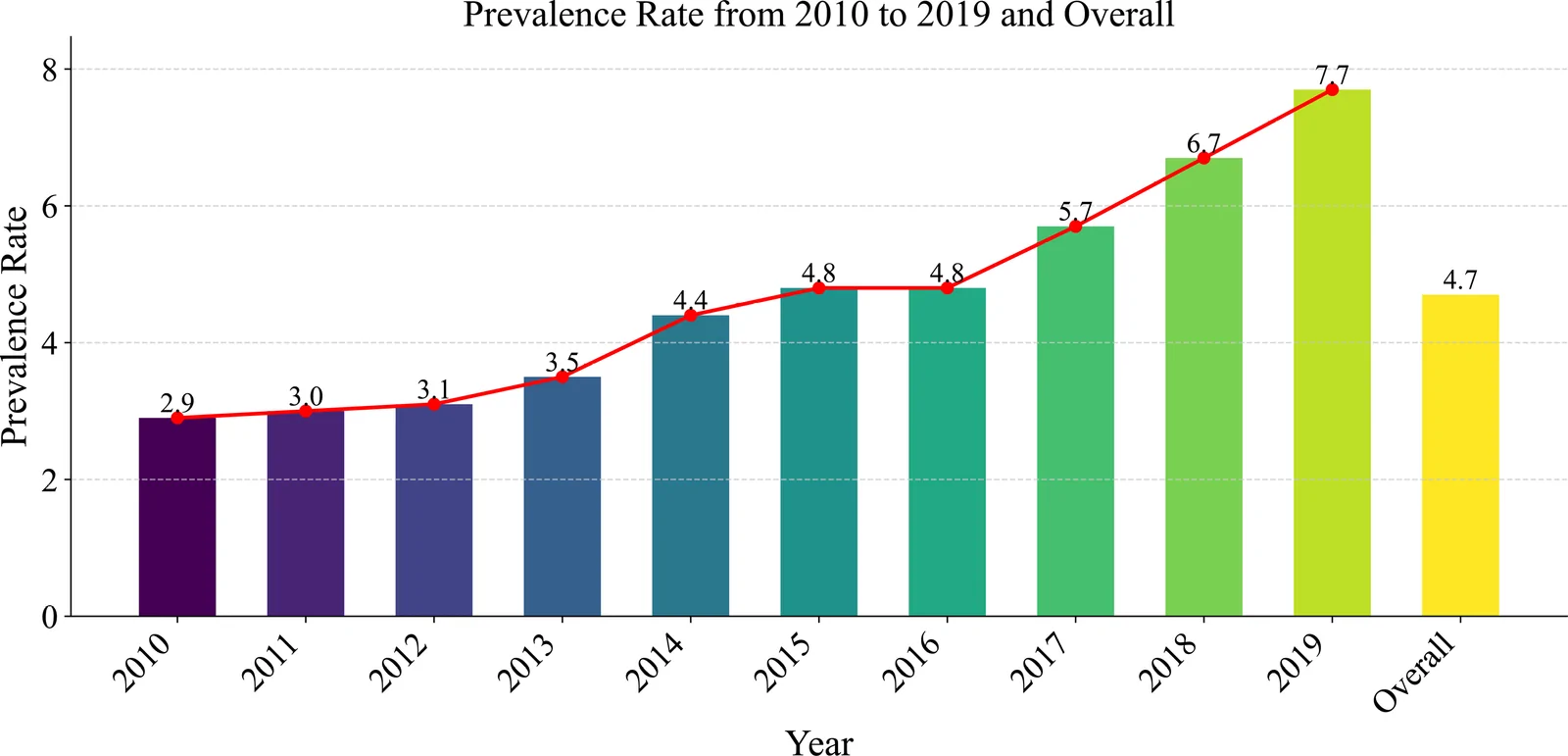
Total annual prevalence of depression and anxiety in the hospitalized birthing population.

During the 10-year study period from 2010 to 2019, a comprehensive analysis of the NIS database revealed a total of 6815,408 birth hospitalizations. Among these, 323,705 hospitalizations had diagnoses of depression and anxiety (Fig. 1). The overall prevalence of perinatal maternal depression and anxiety was 4.7%, with annual prevalence showing a significant upward trend from 2.9% in 2010 to 7.7% in 2019 (Fig. 2).

### 3.2. Demographic characteristics and comorbidities of women in the 2 study cohorts

Table 2 delineates the sociodemographic features of the study population. Women with a diagnosis were a year older, with a median age of 29 years compared to 28 years for those without a diagnosis (*P* < .001) and predominantly White (66.64% vs 48.41%, *P* < .001). They were less likely to be Black, Hispanic, or Asian or Pacific Islander. A higher percentage of women without depression and anxiety had no recorded comorbidities (72.03% vs 15.47%, *P* < .001). Women with depression and anxiety had a longer median length of stay (3 days vs 2 days, *P* < .001) and higher median total charges ($15,500 vs $14,292, *P* < .001). Women with depression and anxiety had higher Medicare coverage (2.29% vs 0.66%) and lower self-pay rates (1.33% vs 2.70%), while Medicaid use was also more common (45.90% vs 44.18%), and private insurance slightly less so (47.43% vs 49.52%, *P* < .001). Women with perinatal depression and anxiety were more commonly treated in large-bed hospitals (56.59% vs 54.84%, *P* < .001) and teaching hospitals (68.61% vs 59.99%, *P* < .001), while being less likely to receive care in the South (33.64% vs 40.33%) and West (20.91% vs 23.72%, *P* < .001).

**Table 2:** Demographic and hospital characteristics of birth hospitalizations with and without depression and anxiety, 2010 to 2019.

Characteristics	Depression and anxiety	No depression and anxiety	*P*
Total (n = count)	323,705	6491,703	–
Total incidence (%)	4.7%		–
Age (median, yr)	29 (24–33)	28 (24–32)	<.0001
Age group, yr			
12–18	9083 (2.81%)	221,363 (3.41%)	<.0001
19–24	73,884 (22.82%)	1610,107 (24.80%)	
25–29	91,834 (28.37%)	1863,834 (28.71%)	
30–34	90,046 (27.82%)	1742,515 (26.84%)	
35–39	47,581 (14.70%)	854,673 (13.17%)	
40–55	11,270 (3.48%)	199,168 (3.07%)	
≥56	7 (0.00%)	43 (0.00%)	
Race			
White	215,712 (66.64%)	3142,535 (48.41%)	<.0001
Black	36,524 (11.28%)	937,655 (14.44%)	
Hispanic	33,195 (10.25%)	1322,528 (20.37%)	
Asian or Pacific Islander	5022 (1.55%)	354,157 (5.46%)	
Native American	2398 (0.74%)	46,425 (0.72%)	
Other	30,854 (9.53%)	688,403 (10.60%)	
Number of comorbidities			
0	50,077 (15.47%)	4675,881 (72.03%)	<.0001
1	130,447 (40.30%)	1186,587 (18.28%)	
2	81,206 (25.09%)	477,071 (7.35%)	
≥3	61,975 (19.15%)	152,164 (2.34%)	
LOS (median, d)	3 (2–3)	2 (2–3)	<.0001
TOTCHG (median, US$)	15,500 (10,217–24,099)	14,292 (9441–21,815)	<.0001
Type of insurance			
Medicare	7411 (2.29%)	42,756 (0.66%)	<.0001
Medicaid	148,577 (45.90%)	2867,859 (44.18%)	
Private insurance	153,520 (47.43%)	3214,720 (49.52%)	
Self-pay	4298 (1.33%)	174,972 (2.70%)	
No charge	159 (0.05%)	6228 (0.10%)	
Other	9740 (3.01%)	185,168 (2.85%)	
Bed size of hospital			
Small	52,985 (16.37%)	1010,470 (15.57%)	<.0001
Medium	87,528 (27.04%)	1921,136 (29.59%)	
Large	183,192 (56.59%)	3560,097 (54.84%)	
Elective admission			
Nonelective	162,867 (50.31%)	3231,745 (49.78%)	<.0001
Elective	160,838 (49.69%)	3259,958 (50.22%)	
Type of hospital			
Nonteaching	101,602 (31.39%)	2597,337 (40.01%)	<.0001
Teaching	222,103 (68.61%)	3894,366 (59.99%)	
Location of hospital			
Rural	31,346 (9.68%)	662,310 (10.20%)	<.0001
Urban	292,359 (90.32%)	5829,393 (89.80%)	
Region of hospital			
Northeast	61,726 (19.07%)	1029,511 (15.86%)	<.0001
Midwest	85,391 (26.38%)	1304,246 (20.09%)	
South	108,894 (33.64%)	2618,323 (40.33%)	
West	67,694 (20.91%)	1539,623 (23.72%)	

LOS = length of stay, TOTCHG = total charge.

Compared to perinatal women without depression and anxiety, those with depression or anxiety were more likely to have the following comorbidities: acquired immune deficiency syndrome (0.15% vs 0.05%), alcohol abuse (0.81% vs 0.08%), deficiency anemia (7.37% vs 5.65%), rheumatoid arthritis/collagen vascular diseases (0.59% vs 0.23%), chronic blood loss anemia (8.05% vs 6.68%), congestive heart failure (0.18% vs 0.05%), chronic pulmonary disease (14.01% vs 3.95%), coagulopathy (3.14% vs 2.11%), diabetes with chronic complications (0.59% vs 0.17%), hypertension (6.08% vs 2.65%), hypothyroidism (5.98% vs 2.95%), liver disease (1.53% vs 0.38%), lymphoma (0.02% vs 0.01%), fluid and electrolyte disorders (1.31% vs 0.55%), metastatic cancer (0.01% vs 0.00%), other neurological disorders (4.79% vs 0.89%), obesity (16.21% vs 7.64%), paralysis (0.13% vs 0.04%), peptic ulcer disease excluding bleeding (0.02% vs 0.00%), peripheral vascular disorders (0.07% vs 0.02%), psychoses (12.06% vs 0.41%), pulmonary circulation disorders (0.08% vs 0.03%), renal failure (0.22% vs 0.07%), solid tumor without metastasis (0.08% vs 0.03%), valvular disease (0.58% vs 0.22%), weight loss (0.15% vs 0.04%), and multiple pregnancy (2.12% vs 1.82%), and were less likely to have uncomplicated diabetes (6.30% vs 3.89%) (Table 3).

**Table 3:** Comorbidities among birth hospitalizations with and without depression and anxiety, 2010 to 2019.

Comorbidity	Depression and anxiety	No depression and anxiety	*P*
Acquired immune deficiency syndrome	499 (0.15%)	3436 (0.05%)	<.001
Alcohol abuse	2612 (0.81%)	5285 (0.08%)	<.001
Deficiency anemia	23,845 (7.37%)	366,926 (5.65%)	<.001
Rheumatoid arthritis/collagen vascular diseases	1895 (0.59%)	15,018 (0.23%)	<.001
Chronic blood loss anemia	26,061 (8.05%)	433,656 (6.68%)	<.001
Congestive heart failure	593 (0.18%)	3364 (0.05%)	<.001
Chronic pulmonary disease	45,342 (14.01%)	256,236 (3.95%)	<.001
Coagulopathy	10,162 (3.14%)	137,091 (2.11%)	<.001
Diabetes, uncomplicated	20,390 (6.30%)	252,635 (3.89%)	<.001
Diabetes with chronic complications	1925 (0.59%)	10,897 (0.17%)	<.001
Hypertension	19,643 (6.07%)	172,129 (2.65%)	<.001
Hypothyroidism	19,366 (5.98%)	191,776 (2.95%)	<.001
Liver disease	4959 (1.53%)	24,548 (0.38%)	<.001
Lymphoma	66 (0.02%)	834 (0.01%)	<.001
Fluid and electrolyte disorders	4256 (1.31%)	35,528 (0.55%)	<.001
Metastatic cancer	35 (0.01%)	311 (0.00%)	<.001
Other neurological disorders	15,509 (4.79%)	57,776 (0.89%)	<.001
Obesity	52,467 (16.21%)	495,847 (7.64%)	<.001
Paralysis	420 (0.13%)	2408 (0.04%)	<.001
Peptic ulcer disease excluding bleeding	79 (0.02%)	253 (0.00%)	<.001
Peripheral vascular disorders	231 (0.07%)	1337 (0.02%)	<.001
Psychoses	39,026 (12.06%)	26,470 (0.41%)	<.001
Pulmonary circulation disorders	265 (0.08%)	1969 (0.03%)	<.001
Renal failure	711 (0.22%)	4744 (0.07%)	<.001
Solid tumor without metastasis	244 (0.08%)	2237 (0.03%)	<.001
Valvular disease	1873 (0.58%)	14,253 (0.22%)	<.001
Weight loss	478 (0.15%)	2483 (0.04%)	<.001
Multiple pregnancy	6853 (2.12%)	118,187 (1.82%)	<.001

### 3.3. Association of depression and anxiety with obstetric and neonatal outcomes

**Figure 3.**
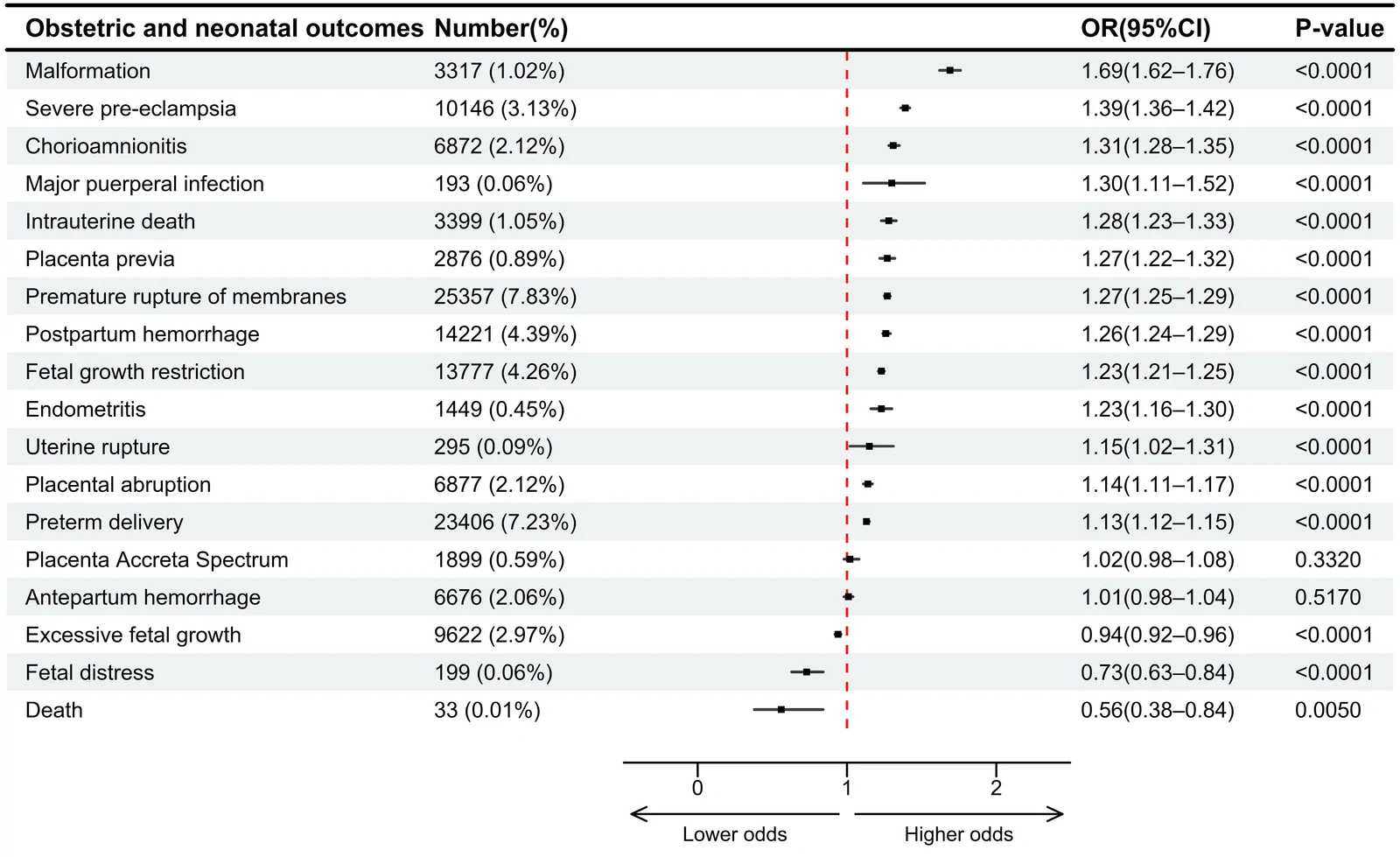
Multivariate Logistic Regression on the association between depression and anxiety and various obstetric and neonatal outcomes, 2010–2019. CI = confidence interval, OR = odds ratio.

In multivariable logistic regression, depression or anxiety diagnoses were associated with higher odds of several obstetric and neonatal outcomes (Fig. 3). In aggregate, both univariate and multivariate statistical analyses revealed substantial correlations between depression and anxiety and a variety of obstetric results. After adjusting the model with the inclusion of multiple variables (Table 1), multivariate logistic regression identified that, compared to the No Depression and Anxiety group, women with depression and anxiety were more likely to experience malformation (OR = 1.69; 95% CI [95% CI] = 1.62–1.76; *P* < .001), severe preeclampsia (OR = 1.39; 95% CI = 1.36–1.42; *P* < .001), chorioamnionitis (OR = 1.31; 95% CI = 1.28–1.35; *P* < .001), major puerperal infection (OR = 1.3; 95% CI = 1.11–1.52; *P* < .001), intrauterine death (OR = 1.28; 95% CI = 1.23–1.33; *P* < .001), placenta previa (OR = 1.27; 95% CI = 1.22–1.32; *P* < .001), PROM (OR = 1.27; 95% CI = 1.25–1.29; *P* < .001), postpartum hemorrhage (OR = 1.26; 95% CI = 1.24–1.29; *P* < .001), FGR (OR = 1.23; 95% CI = 1.21–1.25; *P* < .001), endometritis (OR = 1.23; 95% CI = 1.16–1.30; *P* < .001), uterine rupture (OR = 1.15; 95% CI = 1.02–1.31; *P* < .001), and PA (OR = 1.14; 95% CI = 1.11–1.17; *P* < .001). Preterm birth was also more common in this group (OR = 1.13; 95% CI = 1.12–1.15; *P* < .001). Using the prespecified threshold of *P* ≤ .001, the associations of depression and anxiety with placenta accreta spectrum (OR = 1.02; 95% CI = 0.98–1.08; *P* = .332), antepartum hemorrhage (OR = 1.01; 95% CI = 0.98–1.04; *P* = .517), and maternal death (OR = 0.56; 95% CI = 0.38–0.84; *P* = .005) did not reach statistical significance. Notably, women with depression and anxiety were less likely to experience excessive fetal growth (OR = 0.94; 95% CI = 0.92–0.96; *P* < .001) and fetal distress (OR = 0.73; 95% CI = 0.63–0.84; *P* < .001).

## 4. Discussion

Using the NIS, a large inpatient database, this study examined temporal trends in depression and anxiety diagnoses during birth hospitalizations and their adjusted associations with a broad range of obstetric and neonatal outcomes from 2010 to 2019. In this study, the prevalence rate of depression and anxiety among hospitalized women who give birth increased yearly, from 2.90% in 2010 to 7.70% in 2019, with larger increases in 2014 and after 2017. Two leading perinatal professional organizations issued clinical guidelines in 2015 and 2016 that may be responsible for raising the number of diagnoses of depression and anxiety disorders in 2017.^[19]^ In 2015, the American College of Obstetricians and Gynecologists issued a committee opinion supporting the implementation of universal depression screening for both pregnant women and new mothers, along with ensuring access to proper follow-up care.^[20]^ The following year, in 2016, the U.S. Preventive Services Task Force updated its guidelines for depression screening in adults to include women during pregnancy and in the postpartum period.^[21]^ These recommendations may have contributed to greater recognition and documentation of perinatal depression and anxiety. A recent study also noted that pregnancy may affect women physiologically, psychologically, and socially, and recommended psychosocial health assessment during pregnancy follow-up.^[22]^

The incidence of malformation is significantly higher among women with maternal depression and anxiety. This correlation is supported by a substantial body of research, including a study that highlights the impact of prenatal maternal stress on infant DNA methylation in the first year of life.^[23]^ The association between maternal perinatal stress and depression and infant outcomes is further emphasized by research showing that such stress can lead to alterations in genes related to neurodevelopment, such as *BRD2*, *ERC2*, and *ASF1A.*^[23]^ These genetic changes can potentially influence the risk of neurodevelopmental disorders like autism spectrum disorder and epilepsy, which are often associated with fetal malformations. Our findings, which indicate an OR of 1.69 (95% CI = 1.62–1.76) for malformations, are consistent with previous research.^[24]^

The number of severe preeclampsia cases is significantly higher among women with maternal depression and anxiety. This finding is consistent with previous research showing that mental disorders are more prevalent among pregnant women with severe preeclampsia.^[25]^ Our findings, which indicate an OR of 1.39 (95% CI = 1.36–1.42) for severe preeclampsia, are consistent with previous research that links maternal depression and anxiety with an increased risk of preeclampsia.

Notably, we found that maternal depression and anxiety are significantly associated with chorioamnionitis, with an OR of 1.31 (95% CI = 1.28–1.35). This association is plausible, considering previous research has indicated that a perinatal mood and anxiety disorder diagnosis was associated with a 3.5-fold higher chance of adverse perinatal outcomes, including chorioamnionitis.^[26]^ Chorioamnionitis, which involves an infection of the amniotic fluid and placental membranes, may result in serious complications for both the mother and the fetus, such as preterm labor and neonatal sepsis.^[27]^ Stress and anxiety can have immunosuppressive effects, potentially making pregnant women more susceptible to infections.^[28]^ Additionally, women with depression and anxiety might engage in less healthy behaviors, such as inadequate prenatal care, which could increase their risk of chorioamnionitis.^[29]^

Major puerperal infection is an important postpartum complication. In our study, maternal depression and anxiety were associated with higher odds of major puerperal infection (OR = 1.30; 95% CI = 1.11–1.52). Research supports our findings, showing that dramatic changes in the maternal microbiome due to depression and anxiety may reduce maternal resistance to infection. These mental health conditions may be associated with underlying physiological changes that increase susceptibility to infections.^[30]^ Throughout the prenatal, natal, and postnatal phases, there is a concomitant alteration in maternal physiological processes and internal milieu, resulting in a diminished capacity for self-cleansing within the genital tract and a weakening of maternal immune defences. Particularly during the act of childbirth, the genital tract is in direct contact with the external environment, which facilitates the breaching of the body’s innate protective barriers.^[31]^ Given the identified link between maternal depression and anxiety and the risk of major puerperal infection, it is imperative to enhance infection prevention measures for mothers with these mental health conditions. This could involve targeted education on hygiene practices and closer postpartum monitoring. Additionally, promoting mental health interventions may help strengthen maternal resistance to infections, thereby improving outcomes for both mothers and infants.

Placenta previa, PROM, PA, and preterm birth are all interconnected obstetric complications. Preterm birth is the primary cause of perinatal morbidity and mortality in developed nations. In our study, we observed a notably higher OR for placenta previa (OR = 1.27, 95% CI = 1.22–1.32), PROM (OR = 1.27, 95% CI = 1.25–1.29), and PA (OR = 1.14, 95% CI = 1.11–1.17) among women with maternal depression and anxiety. These conclusions are consistent with preexisting research findings that women with depressive and anxiety symptoms during pregnancy had higher odds of PROM, PA, and preterm birth when compared with women without such symptoms.^[32–35]^ Evidence regarding the association between depression and anxiety diagnoses and placenta previa remains limited; therefore, this finding should be interpreted as hypothesis-generating and requires confirmation in future studies with more detailed clinical timing.

In our study, maternal depression and anxiety were associated with higher odds of intrauterine death (OR = 1.28; 95% CI = 1.23–1.33). Stress and anxiety may affect the hypothalamic-pituitary-adrenal axis,^[36]^ and increased cortisol levels related to hypothalamic-pituitary-adrenal-axis activation may influence uteroplacental blood flow and fetal development.^[37]^ The pathophysiological mechanisms underlying this association are not fully understood, and any explanation based on stress biology remains speculative. Previous studies have often focused on psychological outcomes among women after stillbirth.^[38,39]^ Less is known about whether depression and anxiety diagnoses recorded during birth hospitalization are associated with intrauterine death. This result should be interpreted cautiously because the temporal sequence between psychiatric diagnoses and intrauterine death cannot be determined from NIS codes.

Likewise, the contribution of maternal depression and anxiety to antepartum and postpartum hemorrhage merits close consideration. Postpartum hemorrhage is associated with an increased likelihood of multiple severe complications, encompassing the requirement for blood transfusions, hysterectomy procedures, maternal Intensive Care Unit admissions, sepsis, thrombophlebitis, and potentially, maternal death.^[40]^ In our study, we identified a significant association between maternal depression and anxiety and the occurrence of postpartum hemorrhage (OR = 1.26, 95% CI = 1.24–1.29). A descriptive-analytical study indicates that elevated levels of depression and anxiety during pregnancy are associated with postpartum hemorrhage.^[41]^ Furthermore, many systematic reviews and meta-analyses highlight the increased risk of postpartum depression in women who experience postpartum hemorrhage ^[42,43]^, suggesting a potential bidirectional relationship between these conditions. An association between depression and anxiety and antepartum hemorrhage has not been reported. While our study also did not find a significant association between maternal depression and anxiety and antepartum hemorrhage, this does not negate the possibility of such a connection.

Among the obstetric outcomes evaluated in our study, FGR showed a significant association with maternal depression and anxiety, with an OR of 1.23 (95% CI = 1.21–1.25). The exact pathophysiological mechanisms behind this connection remain unclear. However, it has been proposed that maternal depression and anxiety may influence blood circulation to the uteroplacental region.^[44]^ In fetal medicine, it is well established that adequate placental perfusion is crucial for the fetus to meet its metabolic and circulatory needs.^[45]^ Resistance to placental blood flow, a key marker of hypoxic-ischemic events, is often linked to developmental delays in studies of perinatal brain injury.^[46]^

In our research, we discovered a noteworthy link between prenatal depression and anxiety and the risks of endometritis and uterine rupture, with the OR being 1.23 (95% CI = 1.16–1.30) for endometritis and 1.15 (95% CI = 1.02–1.31) for uterine rupture. It was reported in the literature that an infection of the uterus, such as endometritis, could potentially lead to uterine rupture, and the presence of endometritis is also linked to raised levels of CRP.^[47]^ Previous literature has primarily focused on the elevated risk of postpartum depression and anxiety in women with endometritis compared to those without the condition.^[48]^ However, there is a relative dearth of research exploring the relationship between depression and anxiety and the risk of uterine rupture. Evidence linking depression and anxiety diagnoses with endometritis or uterine rupture is limited; therefore, these findings should be considered exploratory and confirmed in datasets with clearer temporal information.

Placenta Accreta Spectrum was not found to be significantly associated with perinatal depression and anxiety in this study, with an OR of 1.02 (95% CI = 0.98–1.08). This suggests that the development of Placenta Accreta Spectrum may not be closely related to maternal mental health.

In our study, maternal depression or anxiety was associated with lower odds of excessive fetal growth (OR = 0.94, 95% CI = 0.92–0.96) and fetal distress (OR = 0.73, 95% CI = 0.63–0.84). These findings indicate that, counterintuitively, there may be a lower likelihood of these complications in the presence of depression and anxiety during pregnancy. On the 1 hand, previous studies have established that maternal psychological stress and emotional distress are significant risk factors for low birth weight, preterm birth, and intrauterine growth restriction.^[49]^ This suggests that maternal stress could potentially lead to poorer fetal growth, rather than excessive growth. On the other hand, the ORs are close to 1, indicating a small effect size, and further research is needed to confirm these preliminary observations with caution.

Recognizing the limited number of maternal death cases in our study, the absence of a significant association with maternal depression and anxiety does not discount the possibility of a relationship between these factors.

A major strength of this study is the use of a large, nationally representative inpatient dataset, which enabled us to examine associations between documented depression or anxiety diagnoses and a broad range of adverse obstetric and neonatal outcomes. In addition, the use of univariable and multivariable logistic regression analyses allowed us to examine both unadjusted and adjusted associations, while the forest plot provided a clear and concise visual summary of the ORs and their corresponding 95% CIs. Nevertheless, several limitations should be considered. One limitation of this study is the dependence on medical billing data from the NIS, which may introduce inconsistencies in coding.^[50]^ These discrepancies could undermine the accuracy. Another limitation is that this analysis, as a retrospective study using administrative discharge data, cannot establish causality. Depression and anxiety were identified using ICD codes recorded during the same birth hospitalization, and the NIS does not indicate whether these diagnoses were present before delivery or were newly documented after an obstetric complication. This raises the possibility of reverse causation, particularly for postpartum hemorrhage, puerperal infection, and endometritis. Therefore, our findings should be interpreted as demonstrating associations rather than causal effects, and future prospective studies with more precise temporal sequencing of psychiatric symptoms and obstetric events are warranted.

## 5. Conclusion

In this NIS study, depression and anxiety diagnoses were recorded in 4.7% of birth hospitalizations from 2010 to 2019. These diagnoses were associated with higher odds of several adverse obstetric and neonatal outcomes, including malformation, severe preeclampsia, chorioamnionitis, major puerperal infection, intrauterine death, placenta previa, PROM, postpartum hemorrhage, FGR, endometritis, uterine rupture, PA, and preterm birth. Maternal mental health history should be considered when assessing perinatal risk and planning care. These findings also support continued screening, brief intervention, and referral for mental health concerns during prenatal and postpartum care. Future prospective studies may help clarify the clinical timing of these associations.

## Author contributions

**Data curation:** Fufei Zhang, Xinran Zhao, Jingyi Huang, Xiaomin Hou.

**Formal analysis:** Fufei Zhang.

**Software:** Fufei Zhang.

**Visualization:** Xinlin Huang.

**Methodology:** Jingyi Huang.

**Investigation:** Qinfeng Yang, Mengning Dong.

**Supervision:** Xuegao Yu.

**Funding acquisition:** Xiaomin Hou.

**Writing – original draft:** Fufei Zhang, Xinran Zhao, Xinlin Huang.

**Writing – review & editing:** Qinfeng Yang, Xiaomin Hou, Mengning Dong.




