# Supplementary material for: Association between depression and anxiety and adverse obstetric and neonatal outcomes: A retrospective National Inpatient Sample database study
Source: Medicine (Baltimore). 2026 Aug 28;105(35):e50353. doi: 10.1097/MD.0000000000050353 (PMC13529194; doi:10.1097/MD.0000000000050353)
Supplement: Supplementary file 1 [file medi-105-e50353-s001.docx]

**Table S1** International Classification of Diseases, 9th Revision, Clinical Modification (ICD-9-CM) and 10th Revision (ICD-10-CM) diagnosis and procedure codes used to determine birth-related hospitalizations

| Conditions | Code type | ICD-9-CM codes | ICD-10-CM codes |
| --- | --- | --- | --- |
| **Inclusion and exclusion of the study population** | | | |
| **Pregnancy**  **inclusion** | Diagnosis codes | V27, 632, 634, 640, 641, 642, 643, 644, 645, 646, 647, 648, 649, 650, 651, 652, 653, 654, 655, 656, 657, 658, 659, 660, 661, 662, 663, 664, 665, 666, 667, 668, 669, 670, 671, 672, 673, 674, 675, 676, 678, 679 | O601, O602, O80, Z37 |
|  | Procedure codes | 72, 72.2[19], 72.3[19], 72.5[1234], 72.7[19], 74.[01249], 74.99 | 0W8NXZZ, 10D00Z, 10D07Z3, 10D07Z4, 10D07Z5, 10D07Z6, 10D07Z8, 10S07ZZ |
| **Pregnancy**  **exclusion** | Diagnosis codes | 631, 632, 633, 634, 635, 637 | O00, O01, O02, O03, O04, O07 |
|  | Procedure codes | 69.01, 69.51, 74.91, 75.0 | 10A0[034]ZZ, 10A07Z6, 10A07ZX, 10A07ZZ, 10A08ZZ, 10T2[034]ZZ |
| **Drug and alcohol abuse exclusion** | Diagnosis codes | 291, 304 | F10, F111, F112, F12 |
| **Depression and anxiety** | | | |
| **Depression inclusion** | Diagnosis codes | 2962, 2963, 3004, 311, 29690 | F32, F33, F31 |
| **Anxiety inclusion** |  | 3000, 30001, 30002, 30981, 30982, 30983, 30989, 30009 | F411, F419, F40, F93.0, F4310, F4312 |
