# Supplementary material for: Association between depression and anxiety and adverse obstetric and neonatal outcomes: A retrospective National Inpatient Sample database study
Source: Medicine (Baltimore). 2026 Aug 28;105(35):e50353. doi: 10.1097/MD.0000000000050353 (PMC13529194; doi:10.1097/MD.0000000000050353)
Supplement: Supplementary file 2 [file medi-105-e50353-s002.docx]

**Table S2** International Classification of Diseases, 9th Revision, Clinical Modification (ICD-9-CM) and 10th Revision (ICD-10-CM) diagnosis and procedure codes used to determine adverse obstetric and neonatal outcomes

| Conditions | Code type | | ICD-9-CM codes | ICD-10-CM codes |
| --- | --- | --- | --- | --- |
| **Poor obstetric and neonatal outcomes** | | | | |
| **Antepartum hemorrhage** | Diagnosis codes | 6411, 6413, 6418, 6419 | | O441, O46, O67 |
| **Chorioamnionitis** |  | 65840, 65841, 65843, 65920, 65921, 65923 | | O411、O752 |
| **Endometritis** |  | 6159, 6701 | | O8612, N719 |
| **Excessive fetal growth** |  | 6566, 653 | | O366 |
| **Fetal distress** |  | 6563 | | O68 |
| **Fetal Growth Restriction** |  | 6565 | | O365, Z364 |
| **Intrauterine death** |  | 6564, V271, V273, V274, V276, V277 | | O364, Z371, Z373, Z376, Z377, Z379 |
| **Major puerperal infection** |  | 6700，6708 | | O8689 |
| **Malformation** |  | 740 | | O350, O351, O352, O358 |
| **Placenta Accreta Spectrum** |  | 667, 6567 | | O4321, O4322, O4323 |
| **Placenta previa** |  | 6410, 6411 | | O44 |
| **Postpartum hemorrhage** |  | 6660, 6661, 6662, 6663 | | O72 |
| **Premature rupture of membrane** |  | 6581 | | O42 |
| **Placental abruption** |  | 641 | | O458, O459 |
| **Preterm delivery** |  | 6442 | | O601,P073,P590,P612 |
| **Severe pre-eclampsia** |  | 6425 | | O141 |
| **Uterine rupture** |  | 6650, 6651 | | O710, O711 |
